# Occupational Cancers among Employed Women: A Narrative Review

**DOI:** 10.3390/cancers15041334

**Published:** 2023-02-20

**Authors:** Federica Teglia, Giulia Collatuzzo, Paolo Boffetta

**Affiliations:** 1 Department of Medical and Surgical Sciences, University of Bologna, 40138 Bologna, Italy; 2 Stony Brook Cancer Center, Stony Brook University, New York, NY 11794, USA

**Keywords:** female, women, neoplasms, epidemiology, occupational diseases, occupational exposure, sex factors, women’s health

## Abstract

**Simple Summary:**

This narrative review provides new insights, and it reports the current state of knowledge on occupational cancers among women based on previously published research, particularly in three female working categories: beauticians and hairdressers, farmers, and healthcare workers. Finally, a focus on breast cancer is presented among female workers.

**Abstract:**

The facts that occupational cancer in women is under-investigated, with few in-depth analyses are well known. In recent decades the workforce has changed, with an increasing number of women employed. Therefore, the inclusion of women in occupational cancer studies has become more urgent and feasible than in the past decades. The difficulties to evaluate occupational causes of female gynecologic tumors in most past cohorts and the potential variation in outcome responses between men and women must be taken into consideration. This narrative review discusses women’s occupational cancer as a current area of research, focusing on three groups of workers characterized by peculiar exposure to occupational carcinogens and where women are often employed: beauticians and hairdressers; farmers; and healthcare workers. We discuss the most relevant cancers in each working category, with a particular focus on female breast cancer. In the three industries reviewed in detail, there are some risk factors which may affect primarily women, inducing breast cancer and cervical cancer, as well as risk factors that are carcinogenic in both genders, but whose effects are less well known in women.

## 1. Introduction

For several decades, the available data on occupational cancers in women have been scarce, and researchers have shown that studies on work-related cancer need to expand their focus on women. In fact, occupational cancer studies focused mainly on men despite employment patterns for women changing dramatically, and so should lead to reversing this trend [1]. Since the number of women in the workforce has risen and the type of work has changed, women have become increasingly exposed to potential carcinogens. It is also important to consider that the estimated occupation-attributable fraction for total cancer generally ranged between 2% and 8% [2,3].

While it is reasonable to assume that substances that cause cancer among men are carcinogens also in women, Blair [4] noted a number of reasons why specific studies in women are needed. The first and most obvious reason is that purely/predominantly female cancers (i.e., breast and gynecological cancers) cannot be studied among men or have less relevance as they suffer from small numbers. Second, gender differences in carcinogen potency, in exposure patterns and in disease outcomes following the same exposure suggest there may be gender-specific effects, underscoring the need for studies specifically focused on women. Differences could be present in exposure circumstances and susceptibility to workplace toxins. Furthermore, anatomic and physiologic differences between the sexes can influence metabolism, as well as structure and function of organs involved in the absorption, metabolism and elimination of toxins. The variations in exposures, target tissue doses, metabolism of toxicants can result in different responses of the gender to workplace and also environmental chemicals [5]. Thirdly, differences may arise from heterogeneity in job tasks assignments despite comparable classifications (e.g., lower frequency or level of exposure in the same job as results of different task assignments), which can occur even when job titles are the same [6]. Lastly, the ability of individual studies to detect gender-related differences in risk has been limited by the small number of events, in particular among female workers: assessing differences between genders in the risk of developing occupational diseases depends, therefore, on the availability of high-quality data from large series of cases in both men and women. 

We conducted a narrative review on occupational cancer in women. We reviewed estimates of the burden of occupational cancer in women, evidence on cancer risk in three occupational groups with high prevalence of female workers—namely hairdressers, nurses and agriculture workers—and the evidence on occupational risk factors of breast cancer, the main female cancer. We did not conduct systematic searches of the literature, but we included previous reviews and large-scale studies identified via searches in the PubMed database for the years 2000–2022, and from the personal archives of one of the authors (PB).

## 2. Estimates of the Burden of Occupational Cancer in Women

Some reviews assessed the inclusion of women in studies of occupational cancer. Zahm et al. reviewed eight journals from 1971 to 1990, reporting that only 35% of articles included examined white women, and 10% non-white women [7]. A subsequent review reported an increasing proportion of articles that assessed the risk of occupational cancer among women, from 39% in 1991–1995 to 62% in 2006–2009, although the articles that examined risk among women only remained around 10% of the total through the entire period [1]. An international conference was held in Reykjavik, Iceland, in 1998 to assess cancer and reproductive risks among working women, and to discuss methodological challenges in occupational studies of women, reporting an advance in the field of research on occupational health among women [8]. 

One of the largest studies on occupational cancer is the Nordic Occupation Cancer Study (NOCCA) [9], which analyzed cancer incidence data by occupational category for the Nordic populations separately for men and women. The study covers the 15 million people aged 30–64 years in the 1960, 1970, 1980/1981 and/or 1990 censuses in Denmark, Finland, Iceland, Norway and Sweden, and the 2.8 million incident cancer cases diagnosed in these people in a follow-up until about 2005. A strength of this study is the high number of women included in the follow up (a total of 7,454,847, a little more than men). In the primary analysis results are reported for 53 occupational categories and one group of economically inactive people. Some of these groups include many women (e.g., nurses), and more detailed analyses would be helpful to better characterize occupational cancer risks in women.

Given the growing number of women in the workforce, resulting in an increasing proportion of women exposed to occupational risk factors, this narrative review aims to address different aspects of occupational cancer among women, with a focus on three categories of female workers who may be exposed to carcinogenic factors: beauticians and hairdressers, farmers, and healthcare workers.

Table 1 shows the results of the NOCCA study divided by the three working categories analyzed afterwards.

## 3. Beauticians and Hairdressers

Barbers, hairstylists, and cosmetologists represent an important occupational group with 1.7 million workers in Europe [10] and more than 620,000 workers in the USA, and its projected increase in employment from 2020 to 2030 is 19%, which is higher than the average of all occupations [11]. The products used by these workers are principally hair preparations, nail care products and, occasionally, skin care products. Exposure to hazardous chemicals which may be contained in colorants, bleaches, shampoos and hair conditioners released during beauticians and hairdressers’ activities from care products puts these professionals at risk of adverse health effects. Hair sprays expose hairdressers to volatile solvents, propellants and aerosols; nail care products contain methacrylate, and formaldehyde (used as antibacterial agent) is also a possible source of exposure [12]. It was previously determined that these compounds could be absorbed through the skin [13]. Direct-acting urinary mutagens was found to be higher in urine of cosmetologists exposed to hair dyes and of those without exposure [14], suggesting that these workers may absorb hair-dye components systemically. 

Hair care products contain more than 5000 chemicals with mutagenic and endocrine disrupting properties in vitro, and their possible role as a carcinogen in animals and humans [15]. For example, para-phenylenediamines is a primary intermediate generally used in the permanent hair color, and it was found to be a powerful skin sensitizer that can induce breast cancer in rats [16]. Over the past years many of the chemicals discovered to be mutagenic and carcinogenic in hair dyes and other beauty preparations have been banned from use in Europe, the United States and other countries. 

Due to the widespread use of these hair and beauty products by barbers, hairstylists, and cosmetologists, even a small increase in the risk of developing cancer, would have important consequences for this population. However, although some studies found an increased risk of cancer among barbers, hairstylists, and cosmetologists [17,18,19], others found no association [20,21]. 

Given the increasing number of women in the workforce it is possible that occupational exposure to hazardous agents, in particular organic solvents, increasingly contributes to breast cancer incidence [22]. Significantly increased risk for breast cancer was observed among pre-menopausal women in barbers and hairdressers (OR = 5.45, 95% CI 1.85–16.0) [23]. Some studies reported an excessive risk among cosmetologists also in post-menopausal women [24,25]. A previous review [24] of occupational studies found limited evidence of an association with employment as a cosmetologist, while a subsequent study found a slight increased risk of 6% for breast cancer among hairdressers compared with other occupations. The NOCCA study reported a breast cancer SIR of 1.06 (95% CI, 1.01–1.10) for female hairdressers [9]. It should be stressed, however, that most of these studies did not control for potential confounders, including reproductive history, hormone use and adiposity.

Regarding hematopoietic cancer, most studies have found elevated risks of all [26], or other lymphopoietic neoplasms [27,28]. A meta-analysis of 59 studies found a risk increase (pooled RR 1.26, 95% CI 1.14–1.38) for hematopoietic neoplasms combined [29]. A significant excess for Non-Hodgkin lymphoma among female hairdressers was found in Denmark (SIR, 1.92; these workers were included, at least in part, in the NOCCA study mentioned above) [30] and Australia [31], but it did not result in other studies [32]. Hodgkin’s disease presented a non-significant excess for female hairdressers in some studies [33,34], while a meta-analysis showed a moderate risk increase (1.25, 95% CI 1.03–1.51) [29].

Excess bladder cancer incidence has been observed in previous studies [18,35,36], and also a meta-analysis concluded that there is strong evidence for an increased risk of bladder cancer among hairdressers (SRR = 1.34, 95% CI 1.28–1.48), in particular for employment lasting 10 or more years (SRR 1.70, 95% CI 1.01–2.88) [37]. A slight decreased tendency of risk for bladder cancer has been observed in the recent years, possibly due to the banning in the use of some aromatic amines as hair dying ingredients after 1980 [38]. It would be useful to determine whether hairdressers working before 1980 and hairdressers working after 1980 have the same risks. To our knowledge, the only study [39] that distinguishes different exposure periods did not present the data for female hairdressers, but only for male, because no increased risk was found for female. An elevated statistically significant SIR (1.24, 95% CI 1.08–1.43) was observed in the NOCCA study among female hairdressers. However, it is important to consider that the lack of adjustment for potential confounders (e.g., tobacco smoking) could change the occupational risk (e.g., some occupations have characteristics that influence the probability of being a smoker). 

Elevated rates of lung cancer were also consistently observed among female hairdressers in cohort studies [27,36,40], and an increase in the risk of lung cancer that rounded to 30% was calculated in a meta-analysis [29]. The risk was higher when the analysis was restricted to female studies (RR 2.36, 95% CI 1.03–5.40). These results should be considered bearing in mind that there is some evidence suggesting more-frequent smoking among hairdressers than in the general population [41,42]. 

Regarding mortality, a study evaluated the mortality patterns among hairdressers, cosmetologist and barbers using 7.2 million death certificates in 24 US states from 1984 to 1995 [26]. Mortality from cancer was significantly elevated among white and black women for all malignant neoplasms (OR = 1.13, 95% CI 1.10–1.17 and OR = 1.15, 95% CI 1.06–1.24, respectively), lung cancer (OR = 1.32, 95% CI 1.25–1.40 and OR = 1.26, 95% CI 1.07–1.47, respectively), and all lymphatic and hemopoietic cancers (OR = 1.15, 95% CI 1.15–1.25 and OR = 1.31, 95% CI 1.05–1.62, respectively). Mortality from stomach (OR = 1.21, 95% CI 1.01–1.45), colon (OR = 1.12, 95% CI 1.03–1.22), pancreas (OR = 1.24, 95% CI 1.11–1.39), breast (OR = 1.10, 95% CI 1.03–1.17), and bladder (OR = 1.36, 95% CI 1.10–1.68) and from non-Hodgkin’s lymphoma (OR = 1.15, 95% CI 1.01–1.31) and lymphoid leukemia (OR = 1.32, 95% CI 1.02–1.71) was also significantly elevated for white women.

The incidence risk was observed to increase for cancers in different anatomical sites, including those presented above. This plurality should be attributed to the existence of multiple exposure pathways including dermatologic, respiratory and systemic ones, but also the common etiology that some cancers share. 

## 4. Agriculture and Farming

Overall, work can create a life condition and an environment which may turn into a risk or a protective factor against cancer [9]. Farmers share a particular health risk profile, where physical activity, outdoor working, reproductive factors (e.g., high number of children, young age at first birth) and lifestyle habits (e.g., low tobacco smoking prevalence) play a role in reducing the risk of certain cancers compared to other groups of the population, while exposure to specific carcinogens and other sociodemographic factors (e.g., low educational level) may lead to an increased risk of other malignancies [9,43]. Indeed, a large range of agricultural activities implies the use of pesticides, as well as the exposure to infectious agents, arsenic and arsenical insecticides, diesel exhaust, sunlight, dust and other environmental pollutants, some of which have been proven to exert a carcinogenic effect [44]. 

These exposures primarily occur through inhalation and dermal contact [45]. 

In many countries, in particular in traditional societies, farming and agriculture are usually family-led activities where men and women are both involved and share a common environment. In addition, agricultural workers, including laborers, comprise many women. Indeed, several studies on agricultural workers included both sexes, allowing an analysis of exposures and cancer risk among women employed in this industry. One example is the prospective cohort of farm owners and agricultural workers in France (AGRICAN) [46]. Boulanger and coauthors analyzed the risk of bladder cancer within this cohort, identifying for women (44% of the total participants) a particularly high risk when belonging to the field-grown vegetable workers category (HR 3.82, 95% CI = 1.58–9.25). This risk corresponded to a more than doubled risk than that of men. Interestingly, these results were found despite the significantly lower proportion of smokers among women (85% vs. 45% of never smokers, *p* < 0.001), where smoking represents the main risk factor of bladder cancer. 

The risk of lung cancer and brain cancer were also investigated within the AGRICAN cohort. The exposure to carbamate insecticides resulted in concern for a smaller proportion of women (around 17%). Meningioma more often occurred in women (69% of the cases) [47]. Another study found a higher risk of CNS tumors in women than in men exposed to fungicide carbamates (60% vs. 30% excess risk) [48,49]. In many cases, women less frequently used pesticides than men, while their exposure mainly derived from the laundering of contaminated clothes [50]. 

The main evidence of cancer in agricultural workers derives from the International consortium of agricultural cohort studies (AGRICOH) [44], including data of 66,394 individuals from France (i.e., the previously mentioned AGRICAN study [46]), US, Norway, South Korea, Denmark and Australia.

The meta-SIRs for overall cancer incidence based on the pooled analysis were 0.84 (95% CI = 0.78–0.91, I^2^ = 95%) for men and 0.77 (95% CI = 0.67–0.88, I^2^ = 81%) for women. In particular, women working in the agricultural sector had a lower risk of breast cancer (meta-SIR = 0.79, 95% CI = 0.71–0.88, I^2^ = 24%) and cervical cancer (meta-SIR = 0.48, 95% CI: 0.33–0.71, I^2^ = 6%) than general population. Conversely, a higher risk of skin melanoma (meta-SIR = 1.18, 95% CI: 1.01–1.38) and multiple myeloma (meta-SIR = 1.27, 95% CI: 1.04–1.54) was observed in women. Despite the overall small number of multiple myeloma cases, the data suggested an excess of multiple myeloma, particularly in female farmers. The authors related these findings to the outdoor farming tasks that are commonly performed by women, including harvesting and re-entry tasks that can increase their exposure of skin to UV radiation and pesticides.

Different studies found an increased risk of lymphohematopoietic cancers in female farmers [51,52,53,54]. The potential mechanisms of pesticides as carcinogens include hormonal disruption, as reported in relation to organophosphorus compounds [45]. Reproductive cancers risks may also be influenced by pesticides exposures, but results on this are still inconsistent. 

Significant evidence on occupational cancer risks across the sexes comes from a paper by Pukkala et al. The authors reviewed the occupational cancer risk in the Nordic countries, based on data from the Nordic Occupational Cancer (NOCCA) project [9]. A significant lower risk of lip cancer was observed among female farmers in Denmark. Moreover, female farmers displayed a reduced risk of laryngeal cancer in the overall cohort (SIR 0.43, 95% CI = 0.30–0.60), as well as lung cancer. Female farmers showed a lower risk of mesothelioma (SIR 0.65, 95% CI 0.43–0.96), breast (SIR 0.78, 95% CI 0.76–0.80), and cervical (SIR 0.60, 95% CI 0.55–0.64).

The same study found increased risk for female farmers in thyroid cancer (1.18, 1.07–1.30), non-Hodkin lymphoma (1.21, 1.00–1.45), and Hodkin lymphoma in both sexes with mildly higher risk in the female sex (1.14, 1.05–1.24).

Overall, a lower risk of cancer was shown in both sexes and was explained as the consequence of lower exposure to industrial carcinogens. The authors also noticed that all of the lowest-risk occupations among the women are typically “male occupations” which require heavy physical activity, suggesting the role of this factor as protective towards cancer development.

## 5. Healthcare Workers

In 2019 about 73% of people employed as healthcare practitioners and technical occupations in the United States were women [55]. Numerous carcinogenic factors, including ionizing and non-ionizing radiation, chemotherapeutic drugs, anaesthetic waste gases and viruses, expose healthcare workers to a risk of developing occupational cancers.

The antineoplastic drugs are used as a treatment for cancer, but also for multiple sclerosis, psoriasis and rheumatoid arthritis. The toxicity and health risks associated with antineoplastic drugs may occur through absorption by direct contact with skin or by inhalation of the aerosolized drugs during the preparation or administration [56]. The International Agency for Research on Cancer (IARC) has classified a number of antineoplastic drugs (e.g., alkylating agents and topoisomerase inhibitors) as known (Group 1) to be carcinogenic to humans [57]. Healthcare workers who prepare therapies or work in areas in which these therapies are prepared are exposed on a repeated basis to these carcinogens. High concentrations of chemotherapeutic agents have been found in the urine of nurses exposed to these drugs during their preparation and administration, but also during the care of patients who previously received chemotherapy [58,59]. A recent meta-analysis [60] found an increased risk of chromosomal aberrations in healthcare workers exposed to antineoplastic drugs, confirming the need to prevent and limit as much as possible this exposure.

The healthcare setting may expose workers to ionizing radiation, through the use of X-rays or radioactive materials used in either diagnosis or treatment of patients as fluoroscopically guided procedures and administration of radionuclides for nuclear medicine procedures. Workplace exposure to radiation usually consists of protracted exposure to low level radiation, which differs from the extensively studied cancer risks associated with a single acute or fractionated high-dose radiation exposure in the Japanese atomic bomb survivors [61]. Epidemiologic studies of medical radiation workers have found excess risks of leukemia, skin and female breast cancer in those employed before 1950, with less evidence of carcinogenic risk subsequently [62].

Studies investigating cancer risk among female healthcare workers (HCW) have mainly focused on nurses [63,64,65,66] and radiologic technologists [66,67]. For nurses, significantly raised proportional registration ratios (PRR) were seen for three cancers: nasopharyngeal, bladder and skin cancers other than melanoma [68]. Breast cancer in particular has been studied in nurses exposed to possible risk factors such as chemical agents, ionizing/non-ionizing radiation and shift work. In the NOCCA study a statistically significant excess of breast cancer incidence was found in all healthcare occupations among women, excluding assistant nurses [9].

The IARC has classified shift work in group 2A of “probable carcinogens to humans”. Exposure to artificial light at night has been hypothesized to increase the risk of breast cancer as a result of a decrease in the secretion of the hormone melatonin and a subsequent increase in circulating estrogens. Various studies reported a relationship between increased breast cancer risk and cumulative years of working on night shifts [69,70]. A qualitative evaluation of seven meta-analyses [71] concludes that there was fairly consistent evidence for a positive association between ever versus never night shift work and breast cancer risk.

An increased risk of leukemia has been found in physicians and nurses occupationally exposed to antineoplastic drugs [72,73]. Mortality due to myeloid leukemia was significantly elevated among pharmacists (MOR 2.0, 95% CI 2.8–4.6) and clinical laboratory technologists (MOR 2.3, 95% CI 1.5–3.4) [74]. Evidence of elevated risk of leukemia (excluding chronic lymphocytic leukemia) has been found in a cohort of 71,894 (77.9% female) US radiologic technologists [75]. Furthermore, occupational studies reported increased leukemia mortality among early radiologists [76,77,78].

HCW are exposed to biological risk through several infections, including carcinogenic microorganisms like HBV, HCV, HIV, HHV-8, HPV, EBV and Helicobacter pylori (Hp) [79,80].

Percutaneous exposure and sharp injuries represent the most common ways of transmission of HBV and HCV infections in the hospital workplace [79]. The longer the seniority as HCW [79], the higher the risk of accidental contamination with biological agents, given the longer exposure to the risk. Percutaneous exposure and sharp injuries represent the most common ways of transmission of HBV and HCV infections in the hospital workplace. The appropriate use of personal protective equipment (PPE), correct handling of biological material and correct performance of risky procedure on the patients, together with the appropriate management of the accident—including early therapies when available (e.g., post exposure prophylaxis with HBIG and the HBV vaccine, which is 85–95% effective [81])—can reduce the development of infection once the worker has been in direct contact with contaminated biological materials.

Overall, 16,000 HCV, 66,000 HBV, and 1000 HIV infections may have occurred in the year 2000 worldwide among HCWs due to their occupational exposure to percutaneous injuries [82]. The fraction of infections with HCV, HBV, and HIV in HCWs attributable to occupational exposure to percutaneous injuries fraction reaches 39%, 37%, and 4.4%, respectively.

The risk of contamination is higher among certain healthcare professionals, such as surgeons and nurses, and socioeconomic settings, such as Asian countries and Africa.

While the prevalence of injury is in the order of 20–30%, the risk of contracting the clinical hepatitis is around 1–6% [81].

A recent review described a wide variation in the prevalence of needlestick injuries in HCW during their entire career, ranging from 32.4% in Ethiopia to 86.2% in China, while the incidence was reported as 0.97 injuries per HCW per year in Kenya, and 2.18 exposures/person-years [83]. The review mentioned a study from Serbia where higher prevalence of needlestick injuries were reported by women in their entire career [84]. No other gender comparisons were reported for this type of occupational injury, limiting the possibility to estimate the risk by sex.

A prospective study conducted in the 90s found a risk of HIV infection 1.5% for a surgeon working 5 years in Zambia, against 0.1% in more developed countries [85]. This trend is still concerning, as according to a study published in 2020, the prevalence of occupational exposure to HIV in the previous year among healthcare workers in Nigeria was 45%, with 63% of the exposed experiencing multiple potential exposures.

The HPV DNA rate in nasal epithelial cells of gynecologists (84% women) performing procedures generating surgical smoke (e.g., electrosurgery) was found to be higher than gynecologists who did not perform such procedures, with a relationship between the risk of infection and the duration of electrosurgery [86]. Indeed, the risk of airborne HPV transmission during ablation procedures has been described in a review and metanalysis by Palma et al. [87] This suggests a risk of workplace acquired HPV infection in certain groups of HCW.

Among the potentially hazardous agents, Hp should be accounted, as linked to gastric cancer and MALT-lymphoma of the stomach [88]. Healthcare professionals, including dentists, have been reported to be at increased risk of infection. Noticeable evidence has been reported by Matzuda and Morizane, who conducted a prospective study collecting longitudinal blood samples tested for Hp antibodies in more than 550 Dental College employees, identifying a risk of new Hp infection equal to 2.68 (95% CI = 0.55–19.7) compared to controls.

Kheyre et al. recently reviewed the occupational risk of occupationally acquired Hp infection, where health professionals were the working category at higher risk of infection, especially considering those working in gastrointestinal disease units. As argued by the authors, a limit in these estimates is accounting for the reference group, which usually falls into occupational groups.

Infectious-related cancers need a long time to develop. While HBV, HCV and HIV are frequently monitored in the occupational setting as accounted as potential hazards in HCW, other infections, including EBV, HPV and Hp, are much more common and are not accounted as potential occupational hazards. Thus, estimating the attributable fraction of cancer related to these additional agents is currently very difficult. The extension of occupational risk factors to other agents such as Hp and HPV may imply their early detection and treatment, besides encouraging the use of PPE and the adoption of hygienic measures in the healthcare setting.

Indeed, all these infective exposures are preventable by applying hygiene measures and following international guidelines for infection control [89].

## 6. Focus on Breast Cancer

Female breast cancer was the most commonly diagnosticated cancer, with an estimated 2.3 million new cases (11.7% of new cases of cancer) and more than 600,000 new deaths in 2020 [90]. Baseline characteristics (e.g., older age, obesity or overweight, family history), reproductive factors (e.g., early menarche, late menopause, late age at first pregnancy and low parity), estrogen and lifestyle (e.g., excessive alcohol consumption and too much dietary fat intake) can increase the risk of breast cancer [91]. Moreover, some occupational exposures are recognized as risk factors for breast cancer, such as ethylene oxide and night shift work, classified in group 1 and group 2A according to the International Agency for Research on Cancer, respectively. Ethylene oxide is a gas used primarily in the production of ethylene glycol and other chemicals that are used in the production of a number of consumer goods; it is also used to sterilize medical equipment. A Swedish study of a cohort of workers exposed to EtO did not find an increase in risk for breast cancer [92]. However, internal analysis after a longer follow-up found significantly increased rate ratios for breast cancer for women in the two upper quartiles of exposure compared to the lower half of exposure (IRR of 2.76 and 3.55, respectively) [93]. Shift work disrupts biological rhythms, decreasing melatonin levels caused by light at night exposure [94]. Since the IARC evaluation, several additional studies, including five cohort studies [95,96,97,98,99] and eleven case-control studies [70,99,100,101,102,103,104,105,106,107,108,109], have been published on shift work in relation to breast cancer risk. In the NOCCA study [9] the highest SIRs were recorded in groups of female workers who, for career reasons, were more likely to postpone their first childbirth, and with high education, such as military personnel (1.57, 95% CI 1.03–2.30), dentists, journalists and physicians, while the SIR was lowest among fishermen (0.69, 95% CI 0.50–0.92), forestry workers, wood workers, gardeners and farmers. Table 2 [18,22,24,26,27,39,40,70,71,109,110,111,112,113,114,115,116] shows some studies investigating the association between breast cancer and hairdressers/beauticians, healthcare workers and agriculture/farmers. 

## 7. Conclusions

In more recent years there has been a transition from men-focused studies to studies designed and conducted specifically to address occupational risks among women. Beyond the three industries reviewed in detail in the previous sections there are other occupational exposures which may affect primarily women. These include risk factors for female cancers such as breast cancer (e.g., ethylene oxide, night shift work) and cervical cancer (e.g., sex workers), as well as cancers which are prevalent in both genders, such as lung cancer (e.g., tobacco workers) and head and neck cancer (e.g., hospitality workers). Moreover, women employed in a specific sector may go through living conditions that shift their risk of certain cancers compared to the general population, e.g., the reduced risk of breast cancer in agricultural workers may be due to the age at first birth; this is not particularly influenced by farming rather than in occupations requiring higher educational levels, which implies long study time, and the higher number of children registered in farmers.

In conclusion, women might be exposed to carcinogens on the workplace, but the amount and quality of available data are in general lesser than for male workers. Investigations among men may not fully characterize the situation in women because of differences in exposure circumstances for the same job title, biologic differences, and distribution of cancers. A less studied aspect of the problem of occupational cancer in women is that of possible differences in risk according to gender following comparable exposure. The ability of individual studies to detect gender-related differences in risk has been limited by a small number of events in individual studies, in particular among female workers: assessing differences between genders in the risk of developing occupational diseases depends, therefore, on the availability of high-quality data from a large series of cases in both men and women.

## 8. Future Directions

Since the epidemiological research on occupational cancers in males has always been quantitatively superior to that reserved for females, we should expand our knowledge of occupational risks among women both through future specific studies on this focus and through more complete analyses where data are already available. In particular, more detailed analyses of occupational studies with small numbers of women relative to the number of men could provide the basis and guide future research. It is necessary to improve the quality of occupational exposure measures in occupational studies of cancer among women, and the methodological investigation must consider information on possible confounding factors. In the future, studies investigating occupational cancers in women are expected to be large in order to achieve desired statistical power for specific exposures.

## Figures and Tables

**Table 1:** Standardized Incidence Ratio (95% CI) of selected cancer sites by occupational category among men and women from Nordic Occupation Cancer Study.

Cancer Site	Lung Cancer		Breast Cancer		Colon Cancer SIR (95% CI)		Liver Cancer		Bladder, Ureter, and Urethra		Leukemia	
Occupational Category	Women	Men	Women	Men	Women	Men	Women	Men	Women	Men	Women	Men
** Healthcare workers **												
Technical workers	0.98 (0.88–1.10)	0.82 (0.80–0.83)	1.24 (1.19–1.29)	1.04 (0.90–1.21)	1.06 (0.97–1.17)	1.09 (1.06–1.11)	0.77 (0.51–1.11)	0.87 (0.82–0.93)	1.08 (0.90–1.28)	1.02 (1.00–1.05)	0.95 (0.77–1.16)	0.98 (0.94–1.02)
Laboratory assistants	0.88 (0.76–1.03)	0.87 (0.75–1.00)	1.21 (1.14–1.28)	0.42 (0.01–2.34)	0.91 (0.78–1.06)	1.01 (0.84–1.22)	0.96 (0.54–1.58)	1.05 (0.60–1.70)	1.05 (0.79–1.35)	1.11 (0.93–1.32)	1.11 (0.83–1.45)	0.92 (0.64–1.28)
Physicians	0.68 (0.52–0.88)	0.53 (0.49–0.58)	1.35 (1.25–1.45)	1.05 (0.52–1.88)	1.07 (0.88–1.29)	1.14 (1.05–1.24)	0.67 (0.24–1.45)	0.90 (0.70–1.15)	0.76 (0.49–1.13)	1.03 (0.94–1.12)	1.08 (0.72–1.55)	0.93 (0.79–1.09)
Dentists	0.61 (0.45–0.81)	0.50 (0.43–0.58)	1.42 (1.31–1.55)	1.20 (0.39–2.80)	1.08 (0.89–1.30)	1.12 (0.98–1.28)	0.97 (0.44–1.83)	0.98 (0.65–1.42)	0.71 (0.44–1.08)	1.10 (0.96–1.26)	0.79 (0.47–1.23)	1.18 (0.92–1.48)
Nurses	0.69 (0.65–0.74)	0.40 (0.19–0.73)	1.18 (1.15–1.20)	2.29 (0.06–12.75)	0.99 (0.94–1.04)	1.14 (0.67–1.80)	0.92 (0.77–1.09)	0.83 (0.10–2.99)	1.00 (0.92–1.10)	0.72 (0.36–1.29)	0.97 (0.88–1.07)	0.28 (0.03–0.99)
Assistant nurses	1.03 (0.98–1.08)	0.86 (0.74–1.00)	0.95 (0.93–0.97)	0.73 (0.09–2.63)	0.99 (0.94–1.03)	0.95 (0.79–1.15)	0.94 (0.81–1.10)	0.98 (0.58–1.54)	1.08 (1.00–1.17)	1.28 (1.09–1.49)	1.04 (0.95–1.13)	0.82 (0.57–1.14)
“Other health workers”	0.79 (0.73–0.84)	0.83 (0.76–0.90)	1.14 (1.11–1.17)	1.40 (0.70–2.50)	0.99 (0.93–1.05)	0.97 (0.88–1.08)	0.99 (0.79–1.21)	1.01 (0.77–1.30)	1.07 (0.96–1.18)	1.00 (0.90–1.11)	1.04 (0.93–1.17)	0.98 (0.82–1.17)
** Beauticians **												
Hairdressers	1.30 (1.19–1.42)	1.22 (1.12–1.33)	1.06 (1.01–1.10)	1.26 (0.51–2.60)	1.08 (1.00–1.18)	1.06 (0.94–1.19)	1.15 (0.86–1.50)	1.43 (1.08–1.85)	1.24 (1.08–1.43)	1.31 (1.18–1.45)	0.86 (0.71–1.04)	0.86 (0.68–1.09)
** Agricultural workers **												
Farmers	0.46 (0.44–0.49)	0.56 (0.55–0.57)	0.78 (0.76–0.80)	0.83 (0.72–0.94)	0.87 (0.84–0.90)	0.76 (0.75–0.78)	0.66 (0.57–0.77)	0.47 (0.45–0.50)	0.66 (0.62–0.72)	0.68 (0.67–0.70)	0.99 (0.92–1.06)	1.00 (0.97–1.03)
Gardeners	0.54 (0.51–0.58)	0.68 (0.66–0.71)	0.76 (0.74–0.78)	0.95 (0.73–1.22)	0.88 (0.84–0.91)	0.81 (0.78–0.85)	0.83 (0.74–0.93)	0.66 (0.59–0.73)	0.76 (0.71–0.82)	0.77 (0.74–0.81)	1.03 (0.96–1.11)	0.97 (0.91–1.04)

**Table 2:** Examples of studies on the association of occupational exposure and breast cancer risk among selected occupational categories.

Working Sector	Study	Type of Risk Factor	SIR, 95% CI
** Hairdressers and beauticians **			
	Lamba AB et al., 2001 [26]	Organic solvents	1.10, 95% CI 1.02–1.17 (white women)
	Labrèche FP et al., 1997 [22]		5.45, 95% CI 1.85–16.0
	Koening et al., 1991 [115]		3.0, 95% CI 1.1–7.8 (beauticians employed 5 or more years)
	Pukkala E et al., 1992 [40]		1.24, 95% CI 0.97–1.57
	Calle EE et al., 1998 [116]		1.02, 95% CI 0.62–1.69
	Czene K et al., 2003 [39]		1.02, 95% CI 0.95–1.09
** Agriculture and farming **			
	Mills PK et al., 2019 [110]	Organochlorine, organophosphate chemicals	2.25, 95% CI 0.89–7.25
	Settimi L et al., 1999 [114]		0.4, 95% CI 0.3–0.7
	Sritharan J et al., 2019 [111]		0.72, 95% CI 0.61, 0.84
** Healthcare workers **			
	Jartti P et al., 2006 [113]	Radiation	1.7, 95% CI 1.0–3.1 (physicians)
	Mohan AK et al., 2003 [112]		SMR 1.01, 95% CI 0.9–1.1 (radiologic technologists)
	Wegrzyn LR et al., 2017 [109]	Night shifts	0.95, 95% CI 0.77–1.17 cohort 1 2.15; 95% CI 1.23–3.73 cohort 2
	Lie JAS et al., 2011 [70]		1.8, 95% CI 1.1–2.8
	Pahwa M et al., 2018 [71]		Five meta-analyses reported pooled ES for ever/never night shift work exposure; these ranged from 0.99 [95% CI 0.95–1.03, N = 10 cohort studies) to 1.40 (95% CI 1.13–1.73, N = 9 high quality studies)
	Sritharan J et al., 2019 [111]	General reference to hospital-related exposures	1.19, 95% CI 1.14–1.24
